# Probing the drivers of *Staphylococcus aureus* biofilm protein amyloidogenesis and disrupting biofilms with engineered protein disaggregases

**DOI:** 10.1128/mbio.00587-23

**Published:** 2023-05-17

**Authors:** Matthew K. Howard, Karlie R. Miller, Brian S. Sohn, Jeremy J. Ryan, Andy Xu, Meredith E. Jackrel

**Affiliations:** 1 Department of Chemistry, Washington University, St. Louis, Missouri, USA; University of Washington, Seattle, Washington, USA

**Keywords:** biofilm, phenol-soluble modulins, MRSA, disaggregase, amyloid

## Abstract

Phenol-soluble modulins (PSMs) are the primary proteinaceous component of *Staphylococcus aureus* biofilms. Residence in the protective environment of biofilms allows bacteria to rapidly evolve and acquire antimicrobial resistance, which can lead to persistent infections such as those caused by methicillin-resistant *S. aureus* (MRSA). In their soluble form, PSMs hinder the immune response of the host and can increase the virulence potential of MRSA. PSMs also self-assemble into insoluble functional amyloids that contribute to the structural scaffold of biofilms. The specific roles of PSM peptides in biofilms remain poorly understood. Here, we report the development of a genetically tractable yeast model system for studying the properties of PSMα peptides. Expression of PSMα peptides in yeast drives the formation of toxic insoluble aggregates that adopt vesicle-like structures. Using this system, we probed the molecular drivers of PSMα aggregation to delineate key similarities and differences among the PSMs and identified a crucial residue that drives PSM features. Biofilms are a major public health threat; thus, biofilm disruption is a key goal. To solubilize aggregates comprised of a diverse range of amyloid and amyloid-like species, we have developed engineered variants of Hsp104, a hexameric AAA+ protein disaggregase from yeast. Here, we demonstrate that potentiated Hsp104 variants counter the toxicity and aggregation of PSMα peptides. Further, we demonstrate that a potentiated Hsp104 variant can drive the disassembly of preformed *S. aureus* biofilms. We suggest that this new yeast model can be a powerful platform for screening for agents that disrupt PSM aggregation and that Hsp104 disaggregases could be a promising tool for the safe enzymatic disruption of biofilms.

**IMPORTANCE:**

Biofilms are complex mixtures secreted by bacteria that form a material in which the bacteria can become embedded. This process transforms the properties of the bacteria, and they become more resistant to removal, which can give rise to multidrug-resistant strains, such as methicillin-resistant *Staphylococcus aureus* (MRSA). Here, we study phenol-soluble modulins (PSMs), which are amyloidogenic proteins secreted by *S. aureus,* that become incorporated into biofilms. Biofilms are challenging to study, so we have developed a new genetically tractable yeast model to study the PSMs. We used our system to learn about several key features of the PSMs. We also demonstrate that variants of an amyloid disaggregase, Hsp104, can disrupt the PSMs and, more importantly, dissolve preformed *S. aureus* biofilms. We propose that our system can be a powerful screening tool and that Hsp104 disaggregases may be a new avenue to explore for biofilm disruption agents.

## INTRODUCTION

Bacteria produce heterogeneous mixtures of proteins, carbohydrates, and DNA that form biofilm matrices in which the bacteria can then become embedded (1). Upon taking up residence in these matrices, the properties of bacteria are transformed from those of a free-living planktonic state to a multicellular, community-based state (2, 3). This communal living markedly improves the ability of bacteria to develop tolerance to antimicrobial agents and other stressors (1, 4). Biofilms are also highly adherent to surfaces, further promoting the physical resilience of the resident communities (1, 4). For instance, to treat catheter-related infections, antibiotics are often used instead of removing the catheter, which can leave biofilms in place (4). While some microbes are killed, a proportion of the microbes deep within the biofilm can survive. These microbes can then acquire resistance and rapidly recolonize the residual biofilm material (4). Eventually, this can give rise to persistent infections and multidrug-resistant strains, such as methicillin-resistant *Staphylococcus aureus* (MRSA) (1, 3 - 6). This suggests that therapeutic approaches relying only on killing microbes may be ineffective and highlights the importance of developing new strategies for eradicating biofilms alongside the development of new antimicrobial agents (4). More broadly, biofilms associated with other microbes are responsible for corrosion, biofouling, and contamination of process water, and decreased quality of drinking water (1, 4). Therefore, improved molecular understanding of biofilms and new strategies for removal of biofilms are key goals.

Proteins play a major role in the structure and function of biofilms, which are formed by a range of microbes (1, 4, 7, 8). Phenol-soluble modulins (PSMs) are the primary proteinaceous component of *S. aureus* biofilms and they contribute heavily to biofilm structure (5). They are also virulent peptides that stimulate inflammatory responses and lyse human cells (8, 9). High expression levels of PSMs are directly correlated with the virulence potential of MRSA (5). Further, *S. aureus psm* deletion mutants display impaired formation of biofilms, while induced expression of *psms* restored characteristic biofilm properties (7).

In *S. aureus*, PSM peptides are encoded at three different locations in the genome. There are four PSMα peptides encoded in the *psmα* operon, two PSMβ peptides encoded in the *psmβ* operon, and δ-toxin (3, 6). In solution, each of the PSMs forms amphipathic α-helices that have surfactant properties, which are thought to be responsible for the cytolytic activity of PSMs (3, 10). This amphipathic character also likely contributes to the tendency of PSMs to aggregate and spread on surfaces (3). In their soluble α-helical form, PSMs can hinder the host immune response by recruiting, activating, and lysing human neutrophils (8). PSMs transition from these soluble α-helices to adopt the β-sheet rich amyloid fold for incorporation into the biofilm matrix (8). Amyloid is highly stable and resistant to conditions such as proteases, denaturants, and boiling temperatures, any of which would denature most proteins (11, 12). Due to the high stability of amyloid and the specific features of the PSMs, it is likely that PSMs contribute heavily to biofilm structure, growth, and resilience, and that disruption of PSMs may destabilize biofilms.

Functional amyloids also play an important role in yeast, as yeast have harnessed prions (infectious amyloids) for adaptive purposes and as protein-based genetic elements (13 - 16). These processes require tight regulation of amyloid formation and deconstruction, which are mediated by the AAA+ ATPase Hsp104 (17). Bacteria also harbor disaggregases from the Hsp100 family; however, these disaggregases, such as ClpB from *E. coli*, are weaker and can only dissolve disordered aggregated proteins but not more stable amyloid proteins (18). Importantly, while globular proteins have highly variable structures, the amyloid fold is relatively conserved (11). Therefore, disaggregases that can dissolve amyloid comprised of one protein may be active against amyloid comprised of other proteins. Indeed, we have demonstrated that yeast Hsp104 can dissolve amyloid and pre-amyloid conformers comprised of many different proteins that are not naturally present in yeast (18). However, this activity was weak and required very high concentrations of Hsp104 (18). We have therefore pioneered approaches to engineer Hsp104 variants with enhanced activity and demonstrated that engineered Hsp104 variants can counter the misfolding of substrates implicated in amyotrophic lateral sclerosis (ALS), Parkinson’s disease (PD), and sarcoma (19 - 26). Due to the conserved nature of the amyloid fold, we hypothesized that these same Hsp104 variants may be capable of also dissolving biofilm-associated amyloids. Dissolution of the amyloid component of biofilms could destabilize these matrices and allow for their removal.

The inherent heterogeneity of biofilms makes them very challenging to study in a tractable way, which has also limited the development of modulators of biofilm formation. We therefore sought to employ yeast to develop a simple model system to overcome these hurdles. Here, we describe a new yeast model for studying the toxicity and aggregation of PSMα peptides. We anticipate that this system has several key features that will enable improved screening for modulators of biofilm formation. We used this system to explore the effects of expression of each of the four PSMα peptides and delineate sequence-specific drivers of PSMα host toxicity and aggregation. We demonstrate that potentiated Hsp104 variants, which can dissolve amyloid comprised of different proteins that aggregate in human disease, can also suppress PSMα toxicity and dissolve PSMα aggregates. Further, we show that a potentiated Hsp104 variant, Hsp104^A503S^, can disassemble preformed *S. aureus* biofilms, suggesting that amyloid disaggregases may be a promising new avenue for the development of biofilm dispersal agents and validating the use of our yeast model system to identify other modulators. We propose that this yeast model system could be a useful platform for further studies of biofilm-associated proteins and suggest that engineered amyloid disaggregases may be a useful tool for the enzymatic disruption of biofilms.

## MATERIALS AND METHODS

### Yeast strains, media, and plasmids

All yeast were WT BY4741, BY4741∆*hsp104,* or WT W303aΔ*hsp104* (*MATa, can1-100, his3-11,15, leu2-3,112, trp1-1, ura3-1,* and *ade2-1*) (27). Yeast were grown in rich medium (YPD) or in synthetic media lacking the appropriate amino acids. Media was supplemented with 2% glucose, raffinose, or galactose. Plasmids harboring the PSMα genes were generated by synthesizing gBlocks (IDT) with flanking Gateway cloning sites. These fragments were inserted into the indicated vector using Gateway cloning to generate PSMα constructs in pAG423GAL-ccdB, pAG423GAL-ccdB-eGFP, pAG425GAL-ccdB-eGFP, pAG303GAL-ccdB-eGFP, and pAG304GAL-ccdB-eGFP (28). Yeast were then transformed with the indicated plasmids. For integrations, pAG303GAL-ccdB-eGFP constructs were linearized with NheI-HF and pAG304GAL-ccdB-eGFP constructs were linearized with MfeI-HF. pAG416GAL-Hsp104 and variants thereof have been previously described (22, 23). Point mutants of PSMα1-4 were generated using Quikchange site-directed mutagenesis (Agilent) and confirmed by Sanger sequencing.

### Yeast transformation and spotting assays

Yeast were transformed according to standard protocols using polyethylene glycol and lithium acetate (29). Hsp104 variants in the pAG416GAL-Hsp104 plasmid were transformed into the indicated strains. For spotting assays, yeast were grown to saturation overnight in raffinose supplemented dropout media at 30°C. Cultures were normalized to an A_600nm_ = 1.5, serially diluted, and spotted in duplicate onto synthetic dropout media containing glucose or galactose. Plates were analyzed after growth for 2–3 days at 30°C. Each experiment was repeated with at least three independent transformations.

### Yeast growth curve assays

Yeast cells were grown overnight in raffinose supplemented dropout media at 30°C, harvested, washed, and resuspended in galactose supplemented dropout media to a final density of OD_600nm_ = 0.8. 5 µL of the cell suspension was added to 195 µL of galactose supplemented dropout media. All samples were prepared in biological triplicates with independent transformations as well as technical triplicates in 96-well polystyrene microplates that were covered with breathe-easy sealing strips. Plates were incubated at 30°C with shaking (283 cpm) on a microplate scanning spectrophotometer (Biotek Epoch2). Cell density was monitored every 10 min at 600 nm.

### Immunoblotting

Yeast were grown and induced in galactose containing medium for 5 h. Cultures were normalized to A_600nm_ = 0.6, 8 mL yeast cells were harvested, treated in 0.1M NaOH for 5 min at room temperature, and cell pellets were then resuspended into 1× SDS sample buffer and boiled for 4 min. Lysates were cleared by centrifugation at 14,000 rpm for 2 min and then separated by SDS-PAGE (4–20% gradient, BioRad), and transferred to a PVDF membrane. Membranes were blocked in Odyssey Blocking Buffer (LI-COR). Primary antibody incubations were performed at 4°C overnight. Antibodies used were anti-GFP monoclonal (Roche Applied Science), anti-Hsp104 polyclonal (Enzo Life Sciences), and anti-PGK monoclonal (Invitrogen). Membranes were imaged using a LI-COR Odyssey FC Imaging system.

### Filter retention assays

Yeast were grown and induced as for immunoblotting above. Following induction, 5 mL of OD_600nm_ = 0.5 cells were harvested and rinsed in sterile water. Yeast were resuspended in 500 µL spheroplasting solution (1.2M D-sorbitol, 0.5 mM MgCl_2_, 20 mM Tris, 50 mM β−mercaptoethanol, 0.5 mg/mL Zymolyase 100T, pH 7.5) for 1 h at 30°C with light shaking. Spheroplasts were pelleted by centrifugation at 500 RCF for 5 min, and the supernatant was discarded. Samples were then resuspended in 100 µL lysis buffer (100 mM Tris, pH 7.5, 500 mM NaCl, 5 mM MgCl_2_, 10 mM BME, 0.5% Triton, and 1% yeast Protease Inhibitor cocktail). Samples were vortexed at high speed for 1 min and then incubated at room temperature for 10 min. Cells were flash frozen in nitrogen. Lysates were thawed at room temperature, 33 µL sample buffer (2X TAE, 20% glycerol, 10% β−mercaptoethanol, and 0.0025% bromophenol blue) was added, and then incubated for 5 min at room temperature. Cellulose acetate membranes were prepared by washing in PBST and then placed on top of a filter paper in a Minifold I 96-well spot-blot array system; 15 µL of each prepared lysate was applied to the cellulose acetate membrane and then washed three times with 200 µL PBST. Membranes were blocked and imaged as described for immunoblotting. To measure total PSM protein, samples were processed for SDS-PAGE followed by immunoblotting. Bound protein was quantified using ImageStudio Lite software (LICOR). The percent of aggregated protein is the ratio of the signal from protein bound to the cellulose acetate membrane divided by the signal from the band on the immunoblot. In experiments investigating the effect of Hsp104 on this ratio, samples were normalized to the appropriate vector control.

### Microscopy

Indicated yeast strains were grown overnight in raffinose dropout media at 30°C. Cells were then collected and resuspended in galactose dropout media at 30°C to induce protein expression for either 5 h or 15 h. Following induction, yeast were stained using either Hoechst dye to visualize nuclei or CellTracker Blue CMAC (7-amino-4-chloromethylcoumarin) to visualize vacuoles and imaged on either a Nikon Eclipse TE-2000-E or Zeiss LSM 880 Airyscan microscope. All imaging was performed live. For confocal imaging, agarose pads were prepared with the appropriate galactose induction media, cells were applied to #1.5 cover glass, and edges were sealed with nail polish. Confocal images were deconvolved using Zen Black software (Zeiss). All images were prepared using ImageJ (NIH). Quantification of circularity, area, and perimeter of each focus or vesicle was performed manually.

### Biofilm disruption assays

Hsp104, Hsp104^A503S^, and Hsp104^DPLA-DWB^ were purified as previously described (22). The activity of all variants was confirmed by luciferase reactivation assay prior to use. Hsc70 and Hdj2 were purchased from Enzo Life Sciences. Proteinase K was from Invitrogen. *S. aureus* SH1000 was a gift from Petra Levin, and the *S. aureus* SH1000 Δ*αβpsm* strain was from Çagla Tükel (8). *S. aureus* was grown to saturation overnight in LB broth at 37°C; 200 μL of culture was added to each well of a 96 well plate, which was then incubated overnight at room temperature. The following day, the wells were washed with water and then treated with Hsp104 (0.5 μM hexamer) supplemented with ATP (5 mM) and an ATP regeneration system (10 mM creatine phosphate, 0.25 μM creatine kinase) in the presence or absence of Hsc70 (0.167 µM) and Hdj2 (0.167 μM) in buffer (25 mM HEPES-KOH, pH 7.4, 150 mM KAOc, 10 mM MgAOc, and 10 mM DTT). Treatment proceeded for 24 h at 37°C. Following treatment, non-adherent cells were removed by washing the plate with sterile water three times. The wells were then stained with crystal violet (1% w/v) for 10 min, washed with sterile water, and allowed to dry overnight. Wells were photographed, and then the dye was solubilized with 200 μL of 95% ethanol. Absorbance was then quantified at 595 nm using a BioTek Epoch 2 plate reader. All experiments were performed with a minimum of two duplicate wells serving as technical replicates, with a minimum of four independent biological replicates for each condition.

## RESULTS

### A yeast model system for studying phenol-soluble modulin (PSM) aggregation

To study the toxicity and aggregation of PSM peptides in a genetically tractable format, we expressed them in the yeast *Saccharomyces cerevisiae*, which has proven to be an excellent system for studying amyloid formation as well as how protein misfolding drives complex human diseases (22, 26, 30 - 37). Yeast models have been developed for studying the misfolding of α-synuclein, Aβ, IAPP, FUS, and TDP-43, in which expression of these proteins in yeast drives phenotypes that closely recapitulate key features of human disease (31 - 35 - 38 - 39 - 39). Screens using these models have identified small molecule and genetic modifiers of protein misfolding implicated in PD, ALS, Alzheimer’s disease, and diabetes (33, 35, 38, 40 - 42). We anticipated that many of the factors that confound the study of biofilms could be overcome through the use of a simple yeast model system, where we could study the intrinsic properties of the PSM peptides and rapidly screen PSM variants. Screening of PSM variants has typically employed solid-phase peptide synthesis, which cannot be carried out in high throughput. However, large collections of PSM variant sequences can be cloned and readily screened in yeast, allowing for probing of the sequence-specific drivers of PSM toxicity and aggregation.

**Fig 1:**
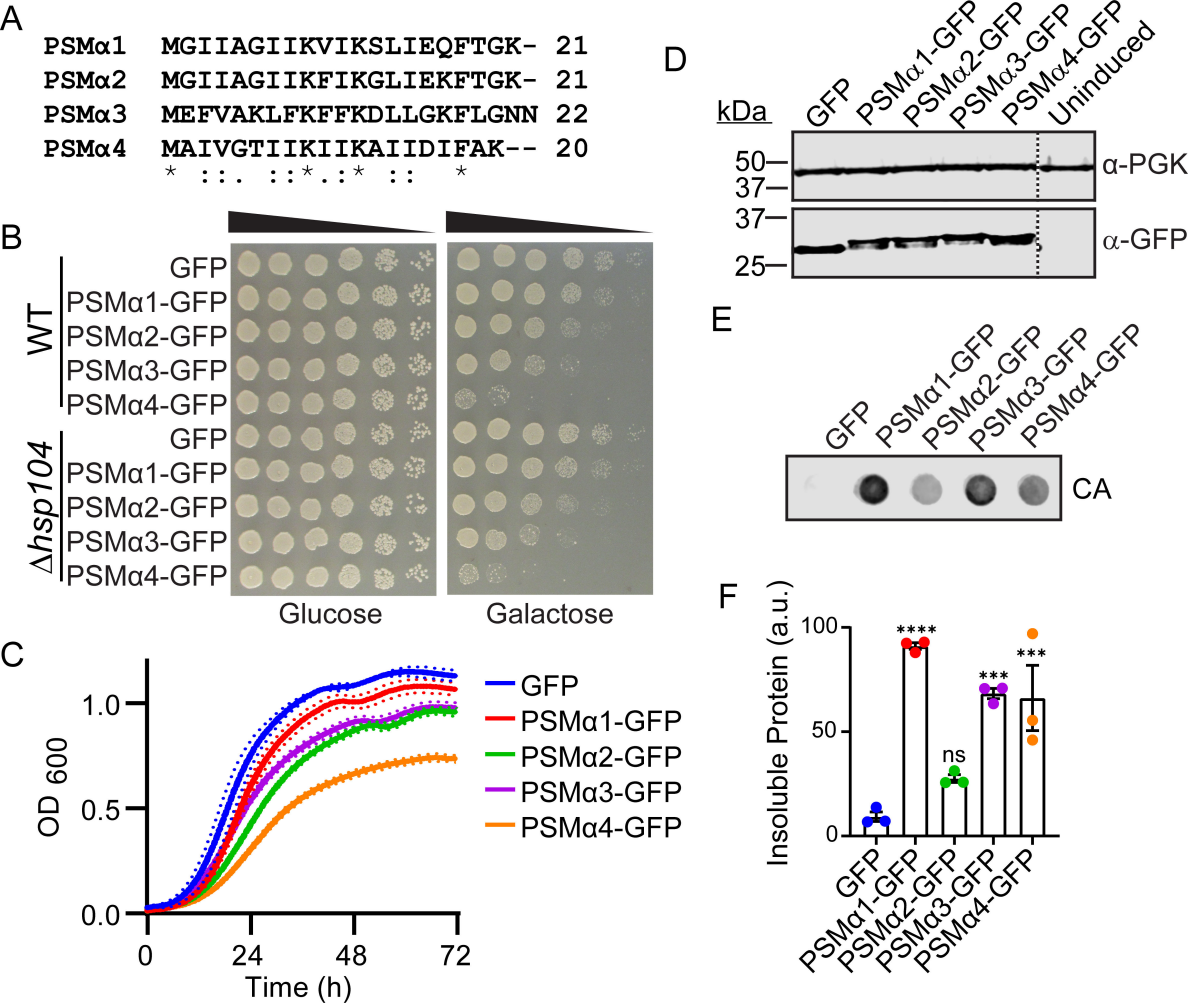
PSMα peptides are toxic and form insoluble inclusions when expressed in yeast. (**A**) A sequence alignment of PSMα1-4 was generated using Clustal Omega. (**B**) BY WT and BYΔ*hsp104* yeast were transformed with the indicated 423GAL-PSMα-GFP plasmid or 423GAL-GFP control. Strains were serially diluted fivefold and spotted on glucose (off) or galactose (on) media. (**C**) BYΔ*hsp104* strains from B were grown in liquid culture and growth was monitored by measuring absorbance at 600 nm over 3 days. Solid lines show the average of three replicates, and hashed lines show ± SEM, *N* = 3. (**D**) BYΔ*hsp104* strains from B were induced for 5 h, lysed, and immunoblotted. Uninduced cells serve as a control. 3-Phosphoglycerate kinase (PGK) serves as a loading control. Hashed line indicates where the blot was spliced for presentation, though all samples were run on the same blot. (**E**) BYΔ*hsp104* strains from B were induced for 5 h, spheroplasted, and lysed. Extracts were then passed over a nonbinding cellulose acetate (CA) membrane. Membranes were then probed using an anti-GFP antibody. Representative results from three biological replicates are shown. (**F**) The ratio of aggregated protein (bound to CA) to total protein (detected via immunoblotting) was calculated. Aggregation of the PSMα proteins were compared to the GFP control using a one-way ANOVA with Dunnett’s multiple comparison test (*N* = 3, individual points shown as dots, bars show mean ± SEM, ***P < 0.001, and ****P < 0.0001).

With this in mind, we chose to study the PSMα peptides, as PSMα peptides are known to be more virulent to the host than the PSMβ peptides, giving us the opportunity to develop a tractable system that could be used for screening for modulators of PSM host toxicity (43). We inserted the PSMα sequences (Fig. 1A) into the pAG423GAL-ccdB-GFP plasmid and generated a series of yeast strains each harboring a single copy of PSMα1-GFP, PSMα2-GFP, PSMα3-GFP, or PSMα4-GFP. We placed PSM expression under control of a tightly regulated galactose-inducible promoter, allowing for inducible expression and passaging without toxicity (28). The C-terminal GFP tag was used in all constructs to improve expression of the short peptides and to allow for their detection by microscopy and immunoblotting. Yeast were then grown to log phase in raffinose-supplemented media and transferred to galactose-supplemented media to induce PSM expression. All four PSMα peptides displayed greater toxicity than a GFP control as monitored by outgrowth on galactose (Fig. 1B). PSMα1 was just modestly toxic, PSMα2 and PSMα3 were moderately toxic, and PSMα4 was highly toxic. To confirm that the GFP tag did not perturb the properties of the PSMα peptides, we also constructed the series of plasmids without the GFP tag (Fig. S1). When expressed without the GFP tag, the PSMα peptides displayed somewhat diminished toxicity, likely because the expression of these very short sequences is weaker. Nonetheless, we observed similar toxicity trends in the presence and absence of the GFP tag, so we can conclude that the tag does not confound our studies. In subsequent experiments, we have used the GFP-tagged constructs to enable detection by microscopy and immunoblotting.

We next aimed to determine if the amyloidogenic PSMs are regulated similarly to endogenous yeast prions. It has been shown that expression of a fragment of the huntingtin protein harboring a polyglutamine expansion is toxic in yeast, but this toxicity is dependent upon the presence of the yeast prion [*RNQ*^+^] and the prion disaggregase Hsp104 (44). We therefore tested the dependence of PSMα toxicity on Hsp104 by comparing toxicity in BYWT and BYΔ*hsp104* yeast. While deletion of Hsp104, which eliminates the [*RNQ*^+^] prion, eliminates the toxic phenotype of polyglutamine (44), the PSMα fusions were similarly toxic in the WT and *hsp104* deletion strains (Fig. 1B). These results suggest that the presence of Hsp104 and its prion propagation activity has no effect on the toxicity of the PSMα peptides. For all remaining experiments, we have restricted our studies to use of the Δ*hsp104* background so that we could later assess the effects of Hsp104 variants on the PSM peptides in the absence of the WT protein. To more quantitatively compare toxicity, we grew the yeast in liquid culture and monitored growth by measuring OD_600_ (Fig. 1C). The trends we observe in liquid culture closely follow those we observe on solid media. Using immunoblotting, we confirmed that toxicity was not simply due to variation in PSMα expression levels (Fig. 1D).

Next, we explored the correlation between toxicity of the PSMα constructs and their solubility. To monitor aggregation of the PSMα peptides in yeast, we implemented a filter retention assay (45). Here, yeast are spheroplasted to allow for gentle lysis. Extracts are then passed over a non-binding cellulose acetate membrane. Soluble proteins pass through this membrane, while insoluble materials will bind the membrane. The membrane can then be probed by immunoblotting, and the signal is normalized to the expression level of the same protein. In the filter retention assay we found that, as expected, GFP did not bind the cellulose acetate membrane (Fig. 1E and F). In contrast, there was a statistically significant increase in binding of PSMα1, PSMα3, and PSMα4 to the cellulose acetate membrane. PSMα2 also displayed a consistent increase in binding to the cellulose acetate membrane, although this difference was not statistically significant. The insolubility we observe correlates with studies suggesting that PSMα peptides form insoluble amyloid or amyloid-like structures (46 - 48). Specifically, it has been shown that while purified PSMα1, PSMα3, and PSMα4 each form insoluble fibrils, under similar conditions, PSMα2 does not fibrillize or form alternative aggregates, although it can undergo seeded assembly to form ThioflavinT reactive species (48). Thus, we conclude that our system can be used to study the toxicity and aggregation of PSMα peptides. Our system recapitulates known features of the peptides, validating our rationale for use of this platform to screen for modulators of PSMα peptide toxicity and aggregation.

### PSMα peptides form vesicle-like structures in yeast

**Fig 2:**
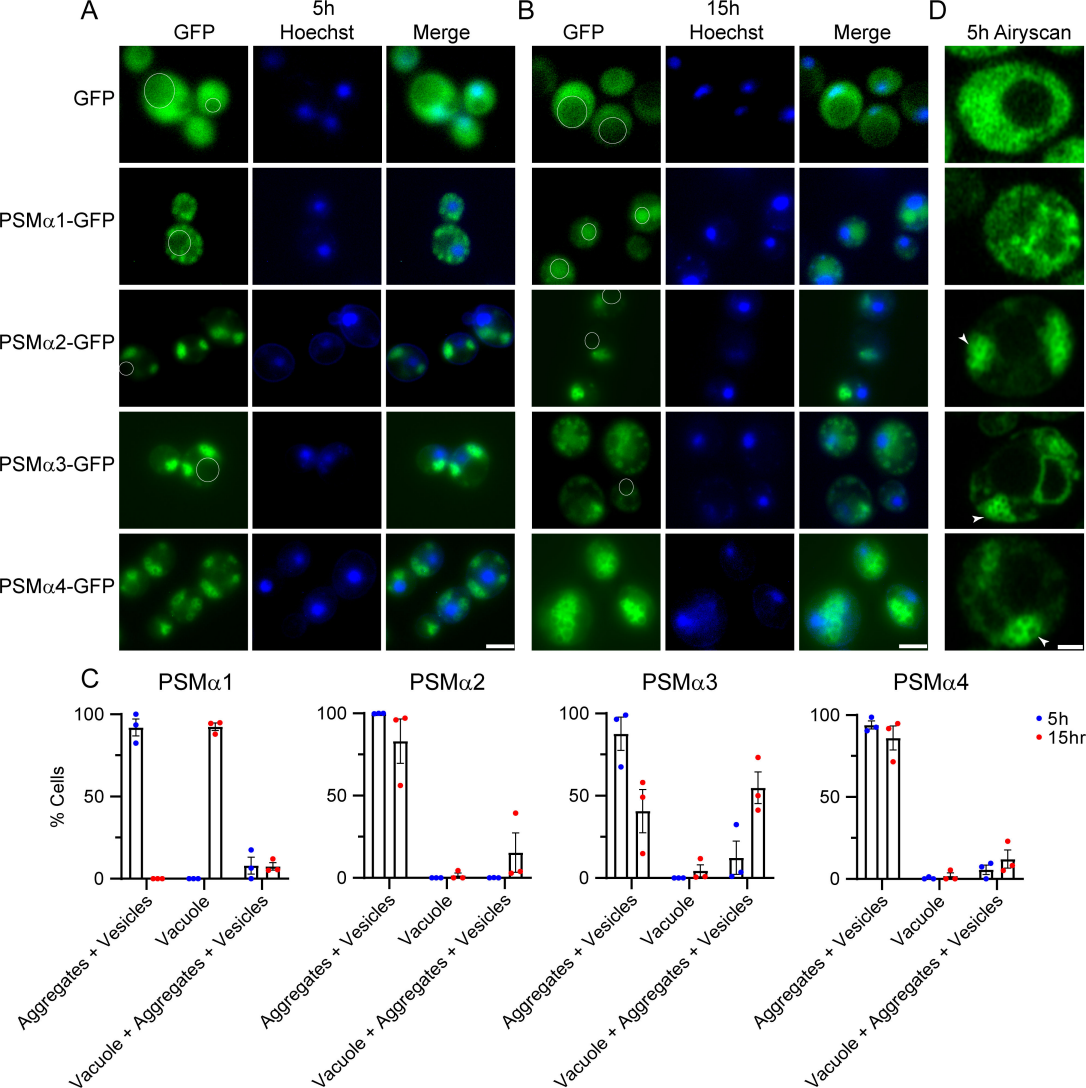
PSMα peptides form foci and vesicle-like structures in yeast. (**A**) BYΔ*hsp104* yeast integrated with two copies of the indicated PSMα peptide or vector control in the 303GAL-PSMα-GFP and 304GAL-PSMα-GFP vectors were analyzed by fluorescence microscopy after 5 h (left) and (**B**) 15 h (right) of induction. Vacuoles, where apparent, are outlined with a white line. Scale bars = 5 microns. See Fig. S2 for images with a vacuole-specific dye. (**C**) Quantification of the microscopy images from A and B. *N* = 3, individual points are shown as dots, and bars show mean ± SEM. (**D**) Superresolution microscopy of the strains after 5 h of induction reveals accumulation of hollow spherical vesicle-like structures. Clusters of vesicles are indicated by white arrowheads. Scale bar = 1 micron. Quantification of puncta and vesicle circularity, area, and perimeter is shown in Fig. S2D to F.

To further characterize the aggregation of the PSMα peptides in yeast, we used fluorescence microscopy. Here, we integrated two copies of each PSMα peptide using the pAG303GAL-ccdB-GFP and pAG304GAL-ccdB-GFP plasmids (28) to ensure similar PSMα expression levels across the entire population of yeast cells of a given strain. We assessed the toxicity of these strains and found that PSMα2, PSMα3, and PSMα4 were all toxic in this format (Fig. S2A). However, toxicity was diminished as compared to the higher copy strains used earlier (Fig. 1), so we did not observe toxicity for the strain expressing PSMα1, though we did detect expression of each of the PSMα proteins, with weaker expression for PSMα1 (Fig. S2B). Upon expression in yeast, we anticipated that if the PSMα peptides aggregated we would observe cytoplasmic foci, while if they did not aggregate we would observe diffuse cytoplasmic fluorescence (Fig. 2). We observed that while GFP alone displayed diffuse fluorescence throughout the cell, appending PSMα1 to GFP led to the accumulation of cytoplasmic foci in nearly all of the yeast cells following 5 h of induction (Fig. 2A and C). We also observed accumulations in nearly all yeast cells upon expression of PSMα2, PSMα3, and PSMα4 (Fig. 2A and C). Strikingly, rather than the small puncta we observe for PSMα1, many of these accumulations appeared to be hollow spheres of protein that we have not previously observed.

To study possible clearance mechanisms of these accumulations over time, we imaged the yeast at both 5 and 15 h following induction (Fig. 2B). For PSMα1, at 15 h following induction we observe few foci, with nearly all of the yeast displaying accumulation of fluorescence in the vacuole (Fig. 2 and Fig. S2C, vacuoles are outlined). The vacuole is a site where misfolded proteins and protein aggregates are typically shuttled for degradation, and transit to the vacuole suggests that PSMα1 is effectively processed for degradation. This transit to the vacuole and consequential increased degradation likely also explains the decreased expression and toxicity noted for PSMα1 (Fig. S2B). In contrast, we observe that the cytoplasmic spherical structures persist at this same 15 h timepoint for strains expressing PSMα2, PSMα3, and PSMα4, although there does seem to be some dispersal of the clusters of spheres. We quantified these effects by counting the number of yeast cells at 5 and 15 h with aggregates and/or vesicles, vacuolar accumulation of GFP, or both (Fig. 2C and Fig. S2C). For PSMα1, following 5 h of induction we find that nearly 100% of cells display aggregates, while following 15 h of induction, nearly 100% of cells display vacuolar PSMα1. Yeast expressing PSMα2 and PSMα4 show little shift in localization over time. Interestingly, PSMα3 shows a more intermediate effect, where a greater subset of cells displays vacuolar accumulation of PSMα3 after 15 h of expression. Although there is notable transit of PSMα3 to the vacuole, these cells also retained some aggregates in the cytoplasm and in vesicles, which cannot be cleared and remain toxic to the cell. Thus, we propose that accumulations of PSMα1 are the most readily cleared from the cell, and this transit of PSMα1 to the vacuole detoxifies PSMα1. In contrast, aggregates and vesicles comprised of PSMα2, PSMα3, and PSMα4 persist, correlating with their enhanced toxicity.

To more closely examine the spherical structures we observed, which were especially apparent for the strain expressing PSMα4, we studied the strains using superresolution confocal microscopy (Fig. 2D). As found previously (Fig. 2A), we observed abundant puncta throughout the cytoplasm for the PSMα1 strain. However, for PSMα2, PSMα3, and PSMα4, we observed clusters of spherical, hollow, vesicle-like structures, which we now refer to as vesicles (Fig. 2D, vesicles are indicated with arrowheads). To quantify the differences in puncta morphology among the four PSMα strains, we calculated the circularity, area, and perimeter of the puncta (PSMα1) or vesicles (PSMα2, PSMα3, and PSMα4). We find that both the PSMα1 puncta and PSMα2, PSMα3, and PSMα4 vesicles are nearly spherical, though the vesicles are somewhat more circular than the PSMα1 puncta (Fig. S2D). The vesicles of PSMα2, PSMα3, and PSMα4 have both a larger area and perimeter as compared to the PSMα1 puncta (Fig. S2E to F). These vesicle structures likely form due to the surfactant properties of the peptides and may be similar to the cytolytic extracellular vesicles secreted by *S. aureus*, where the formation of these vesicles is dependent on the presence of PSMα peptides (49).

### Sequence-specific drivers of PSMα toxicity and aggregation

**Fig 3:**
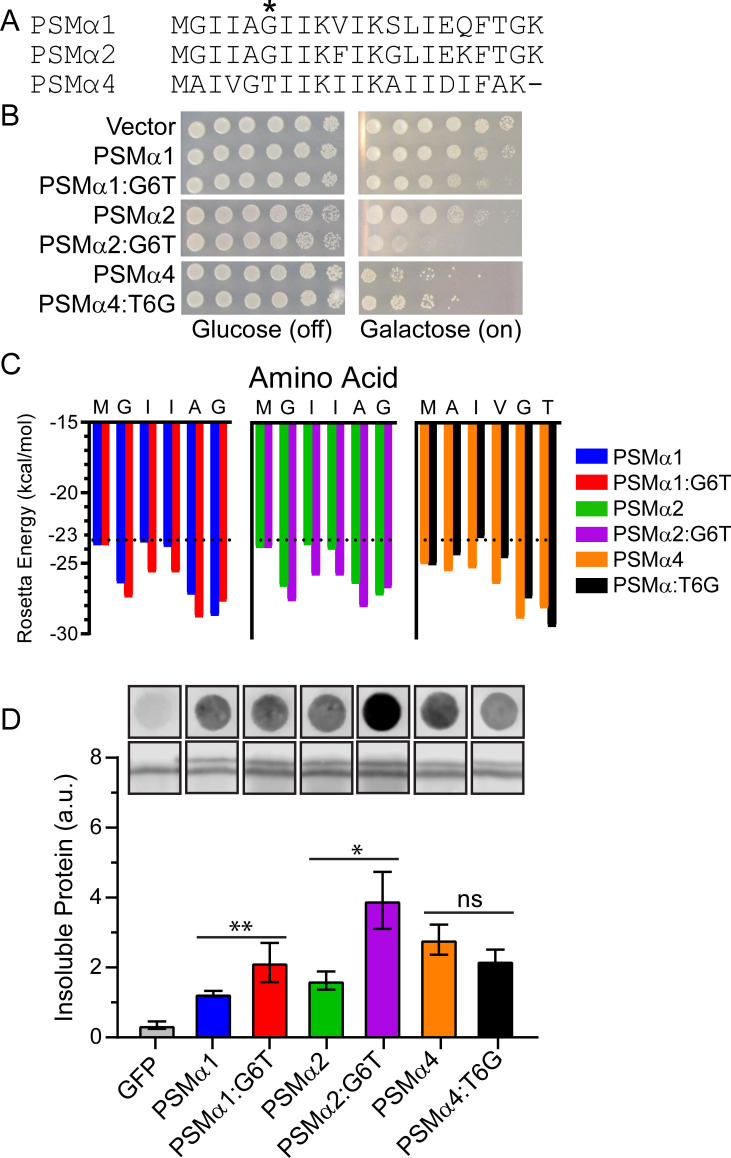
Missense mutations of PSMα residue 6 drive propensity for aggregation, toxicity, and solubility. (**A**) Sequence alignment PSMα1, PSMα2, and PSMα4 indicates lack of conservation at residue 6. (**B**) BYΔ*hsp104* yeast were transformed with the indicated 423GAL-PSMα-GFP plasmid or 423GAL-GFP control. Strains were serially diluted 5-fold and spotted on glucose (off) or galactose (on) media. (**C**) Analysis of the sequences by ZipperDB (50). Steric zippers are predicted to form when the Rosetta energy of a hexapeptide is below the threshold of −23 kcal/mol, which is denoted by a hashed line. (**D**) Strains from B were induced for 5 h, spheroplasted, and lysed. Extracts were then passed over a nonbinding cellulose acetate (CA) membrane. Membranes were then probed using an anti-GFP antibody. Representative results from three replicates are shown (top row). Extracts were also probed via immunoblotting to assess total GFP (second row). The ratio of aggregated protein (top row, bound to CA) to total PSMα-GFP protein (second row, from immunoblotting) was calculated (bottom). Aggregation of each WT PSMα protein was compared to its respective mutant using two-tailed t-tests. (N ≥ 6, bars show mean ± SEM, *P < 0.05, and **P < 0.01). Note that the shown filter retention assay and immunoblotting samples were all run on a single membrane in a randomized order, with a single representative trial shown. Lanes are cropped and re-ordered for presentation purposes.

To define the basis for the differences in toxicity we observed, we next aimed to predict and test how various substitutions would modulate the aggregation and toxicity of the PSMα peptides. To do so, we compared the sequences of PSMα1, PSMα2, and PSMα4 by constructing a sequence alignment using Clustal Omega (Fig. 3A). We excluded PSMα3 from our analysis because it adopts a non-canonical α-helical conformation in the fibrillar form, restricting our ability to make direct comparisons (46). The sequence identity between PSMα1 and PSMα2 is approximately 86%, while the identities between PSMα1 and PSMα4, as well as between PSMα2 and PSMα4, are approximately 45%. Looking for nonconservative mutations, we noted that glycine was present in PSMα1 and PSMα2 at position 6, while threonine is in this position in PSMα4. Aside from this site, conservation at the remaining positions was rather high between the three peptides. We therefore further analyzed the role of glycine and threonine at position 6. We constructed PSMα1^G6T^, PSMα2^G6T^, and PSMα4^T6G^, each in the pAG423GAL-ccdB-GFP plasmid, and assessed toxicity (Fig. 3B). We find that substitution of threonine for glycine in PSMα1 and PSMα2 enhances their toxicity. In contrast, substitution of glycine for threonine in PSMα4 suppresses its toxicity. These results suggest that substitution of glycine for threonine at this position might be a key modulator of the properties of PSMα peptides.

To further explore the effects of these substitutions, we examined these sequences using the program ZipperDB (50). ZipperDB is a structure-based threading algorithm that scores six amino acid segments for propensity to form self-complementary β strands, or steric zippers, which comprise the spine of amyloid fibrils. Rosetta energies are calculated, with a threshold of –23 kcal/mol corresponding to amyloidogenicity. Results from this ZipperDB analysis (Fig. 3C) support the conclusions from our toxicity assays. We observe that for both PSMα1 and PSMα2, the G6T substitution enhances the propensity of this region to form a steric zipper. In contrast, introduction of the T6G substitution in PSMα4 decreases the propensity of this region to form a steric zipper.

To determine if these changes in toxicity correlate with predicted changes in solubility, we again performed filter retention assays (Fig. 3D). The trends that we observe correlate with those from the toxicity assays and the ZipperDB analysis. Relative to their WT counterparts, we observe a mild increase in insolubility for PSMα1^G6T^ and a more robust increase in insolubility for PSMα2^G6T^. We also observe a slight, but not statistically significant, decrease in insolubility for PSMα4^T6G^. These results suggest that this site may be a key modulator of PSMα aggregation and that our system can be used to probe the molecular drivers of PSMα amyloid formation.

### Potentiated Hsp104 variants suppress the toxicity of PSMα peptides

**Fig 4:**
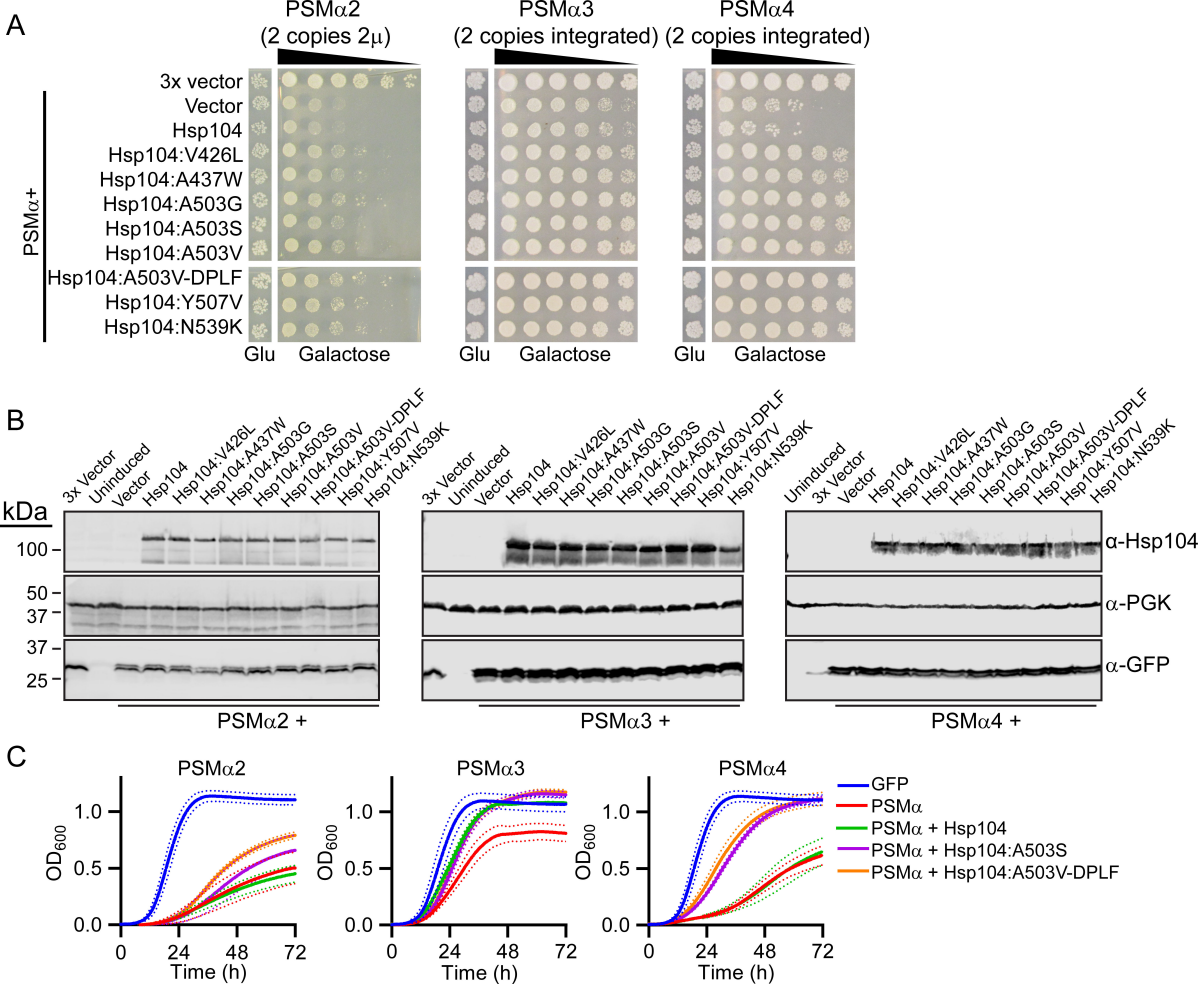
Potentiated Hsp104 variants suppress the toxicity of PSMα peptides. (**A**) BYΔ*hsp104* yeast was transformed with 423/425GAL-PSMα2-GFP while w303Δ*hsp104* yeast was transformed with 303/304GAL-PSMα3-GFP or 303/304GAL-PSMα4-GFP. The resulting strains were transformed with the indicated 416GAL-Hsp104 variant or vector control. Strains were serially diluted fivefold and spotted on glucose (Glu, off) or galactose (on) media. Only the most dilute spots are shown for the glucose control plates. (**B**) Strains from A were induced for 5 h, lysed, and immunoblotted. Uninduced cells serve as a control. 3-Phosphoglycerate kinase (PGK) serves as a loading control. (**C**) Selected strains from A were grown in liquid culture, and growth was monitored by measuring absorbance at 600 nm over 3 days. Solid lines show the average of three biological replicates. Hashed lines show ± SEM.

With a new system for studying the mechanisms and modulators of PSMα toxicity and aggregation in hand, we were next interested in using this system to investigate potential modulators of PSMα aggregation. Previous studies have shown that a diverse range of amyloid and amyloid-like proteins can be solubilized by variants of Hsp104, an AAA+ ATPase protein disaggregase from yeast (22, 26, 51 - 53). While Hsp104 has limited activity against most of these non-native misfolded substrates, we have engineered potentiated variants harboring missense mutations that display robust remodeling activity (18, 22 - 54 - 54). We therefore hypothesized that these same potentiated Hsp104 variants that can remodel substrates, including α-synuclein, TDP-43, and FUS, may also be capable of remodeling PSMα peptides. We coexpressed PSMα2, PSMα3, and PSMα4 with Hsp104 and a series of variants: Hsp104^V426L^, Hsp104^A437W^, Hsp104^A503G^, Hsp104^A503S^, Hsp104^A503V^, Hsp104^Y257F-A503V-Y662F^ (Hsp104^A503V-DPLF^), Hsp104^Y507V^, and Hsp104^N539K^, which represent a range of variants that have been identified to suppress the toxicity, misfolding, and mislocalization of α-synuclein, TDP-43, and FUS (22, 23). To assess the effects of Hsp104 expression on PSMα toxicity and aggregation, we modified our PSMα strains. We found that toxicity of the integrated PSMα2 strain decreased substantially upon introduction of a third plasmid (Fig. S3), so we instead employed a strain expressing two copies from pAG423GAL-PSMα2-GFP and pAG425GAL-PSMα2-GFP (Fig. 4) to drive higher expression of PSMα2 as compared to the single copy strain used in Fig. 1. We also used this higher expression format for testing Hsp104 variants against PSMα1 (Fig. S3), which is also only weakly toxic when integrated (Fig. S2A). For PSMα3 and PSMα4, we employed the strains with two integrated copies of each PSMα peptide (Fig. 2 and Fig. S2A).

Hsp104 does not suppress the toxicity of α-synuclein, TDP-43, or FUS in yeast (22), and we similarly find that Hsp104 does not suppress the toxicity of PSMα2, PSMα3, or PSMα4 (Fig. 4A). In contrast, each of the potentiated Hsp104 variants tested robustly suppresses the toxicity of PSMα2. The strain integrated with PSMα3 did not display very high toxicity but we still observe a strong rescue by each of the variants. PSMα4 toxicity was more robust than PSMα3, and we again observe that each of the potentiated variants tested strongly suppresses this toxicity, restoring growth to a level similar to that of yeast expressing no PSMα4. Against PSMα1, we observed no rescue by Hsp104 variants (Fig. S3). To confirm that these changes are not simply due to altered expression levels, we measured expression of the PSMα peptides and each of the Hsp104 variants, and we observe consistent expression levels for each strain (Fig. 4B and Fig. S3). To further assay these changes in toxicity we monitored growth in liquid culture for two of the better characterized variants, Hsp104^A503S^ and Hsp104^A503V-DPLF^. We observe trends similar to those from the spotting assays. Again, Hsp104 does not suppress PSMα2 toxicity while the two potentiated variants robustly counter this toxicity. Against PSMα3, the two potentiated variants and Hsp104 have similar effects in suppressing toxicity relative to PSMα3 alone. Against PSMα4, we note robust toxicity suppression by both Hsp104^A503S^ and Hsp104^A503V-DPLF^, while Hsp104 does not modify PSMα4 toxicity (Fig. 4C). Thus we conclude that Hsp104 variants can suppress the toxicity of PSMα proteins, which also validates use of this model for screening for modulators of biofilm protein amyloidogenesis.

### Potentiated Hsp104 variants counter PSMα aggregation and vesicle formation

**Fig 5:**
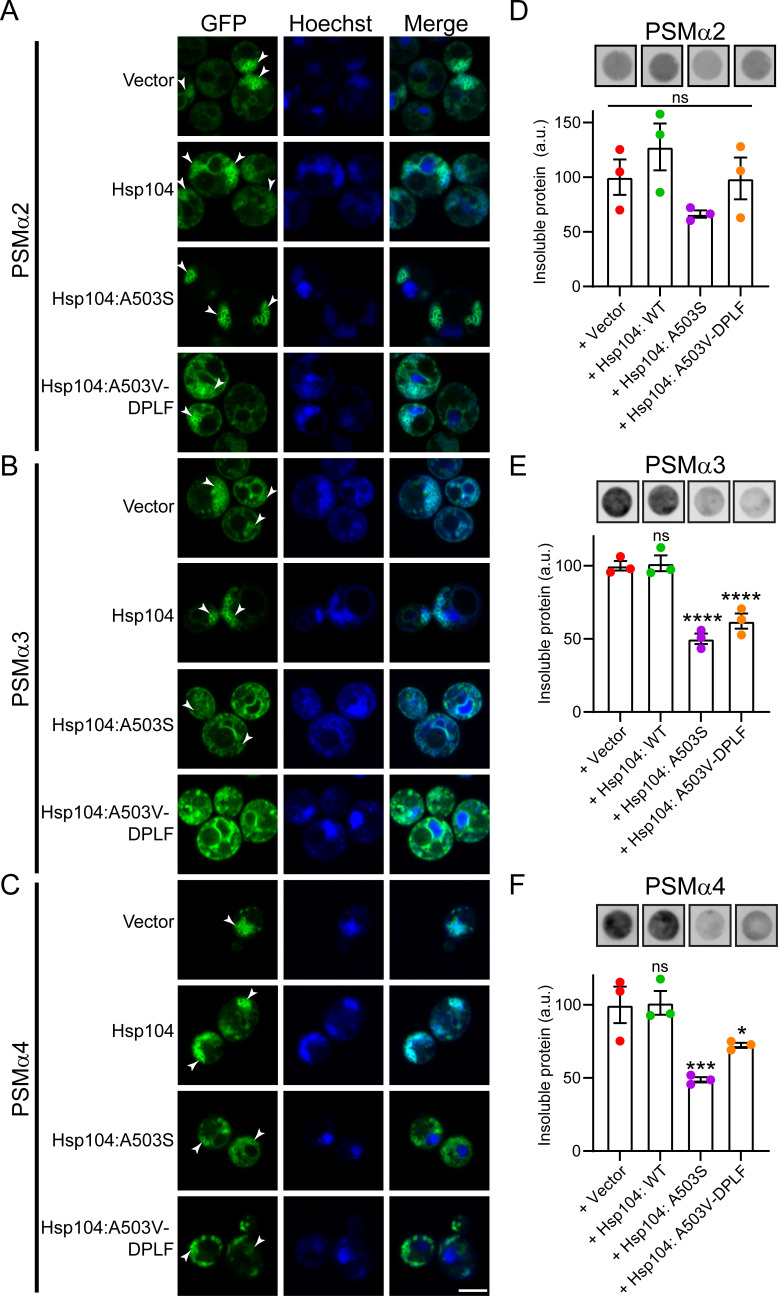
Potentiated Hsp104 variants disrupt PSMα vesicle structures and solubilize PSMα proteins. (**A**) BYΔ*hsp104* yeast integrated with 303/304GAL-PSMα2-GFP were transformed with the indicated 416GAL-Hsp104 variant or 416 GAL vector control. Strains were induced for 5 h, stained with Hoechst to visualize nuclei (blue), and imaged. (**B**) Experiments were performed as in A but with PSMα3. (**C**) Experiments were performed as in A but with PSMα4. Vesicles are indicated by arrowheads. Scale bar = 3 microns. (**D**) Strains from A were processed for a filter retention assay following 5 h of induction. CA membranes were probed using an anti-GFP antibody. Representative results from three replicates are shown (top). The ratio of aggregated protein (bound to CA) to total PSMα-GFP protein was calculated. (bottom). (**E**) Experiments were performed as in D but with PSMα3. (***F***) Experiments were performed as in D but with PSMα4. For D-F *N* = 3, individual points are shown as dots, and bars show mean ± SEM. Aggregation of the PSMα proteins when coexpressed with Hsp104 and Hsp104 variants were compared to the GFP control using a one-way ANOVA with Dunnett’s multiple comparison test, *P < 0.1, ***P < 0.01, ****P < 0.001.

We next aimed to determine if the Hsp104 variants clear PSMα aggregates and vesicles in yeast. To do so, we performed superresolution fluorescence microscopy following 5 h of induction. For PSMα2, we observed the accumulation of clusters of vesicle-like structures that are not cleared by Hsp104 (Fig. 5A, vesicles denoted with arrowheads). Upon expression of Hsp104^A503S^, these vesicles appear larger and less spherical. Upon expression of Hsp104^A503V-DPLF^, fewer vesicle-like structures were noted. These results correlate with the results of the toxicity assays, where Hsp104^A503V-DPLF^ more robustly suppresses toxicity as compared to Hsp104^A503S^, suggesting that clearance of these structures alleviates their toxicity. We next monitored the strains expressing PSMα3 (Fig. 5B). We again find that the accumulations of vesicle-like structures of PSMα3 are not modified by Hsp104. However, both of the potentiated variants clear these structures. Finally, we performed these experiments with PSMα4 (Fig. 5C) and again find that vesicle-like structures accumulate and persist upon expression of Hsp104. Upon expression of the two potentiated variants, vesicle-like structures remain. However, they appear to have altered morphology and are less clustered, suggesting they are being more subtly remodeled.

To determine if these changes are due to solubilization of the accumulations, we again employed filter retention assays. We find that PSMα2 remains primarily soluble in yeast (Fig. 1E and F; Fig. 5D). Nonetheless, Hsp104^A503S^ and Hsp104^A503V-DPLF^ subtly solubilize PSMα2, though these effects are not statistically significant. Against PSMα3, both potentiated variants solubilize approximately 50% of the aggregated material (Fig. 5E). Finally, we find that both Hsp104^A503S^ and Hsp104^A503V-DPLF^ solubilize PSMα4, clearing ~50% and 25% of PSMα4, respectively (Fig. 5F). Thus, we conclude that potentiated Hsp104 variants can eliminate PSMα vesicles and solubilize PSMα protein.

### A potentiated Hsp104 variant, Hsp104^A503S^, can disassemble *S. aureus* biofilms

**Fig 6:**
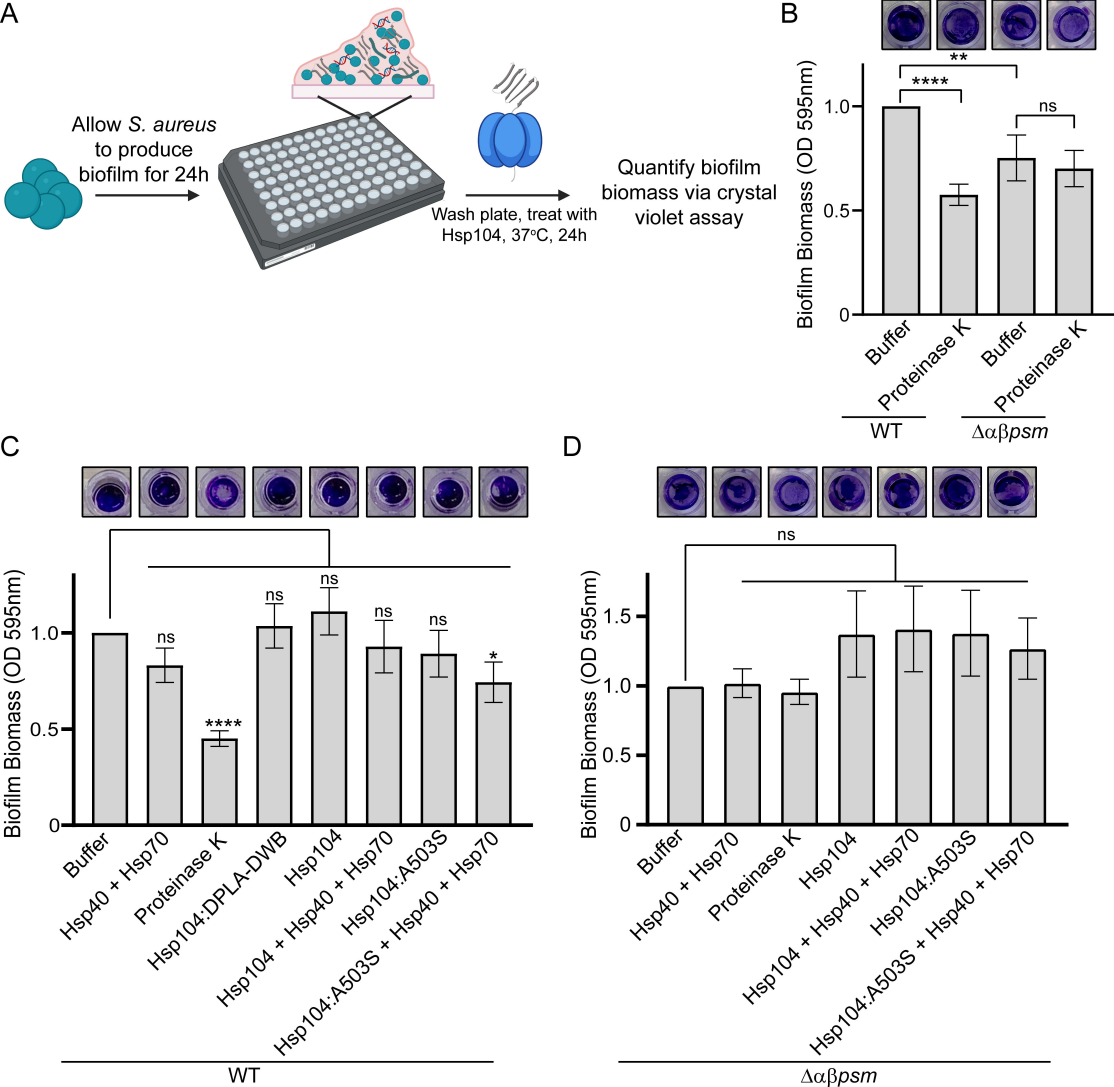
Hsp104^A503S^ disrupts *S. aureus* biofilm formation. (**A**) Schematic depicting the experimental design was created using BioRender.com. (**B**) SH1000 WT or SH1000 Δ*αβpsm S. aureus* was incubated in 96 well plates for 24 h to allow biofilm biomass to accumulate. Following washing, residual biomass was stained with crystal violet dye to visualize biomass. Images (top) show wells that are all from a single trial. Wells were then resuspended and quantified. Biomass for each condition was compared to the treatment of SH1000 *S. aureus* control treated with buffer using a two-tailed, unpaired t-test. (*N* ≥ 6 biological replicate trials, with three technical replicate wells for each trial, bars show mean ± SEM, **P < 0.01, ****P < 0.0001, and ns *P* > 0.05). (**C**) Experiments were performed as in B using SH1000 WT *S. aureus*. Here, following washing, residual biomass was treated with buffer, Hsp104^WT^, Hsp104^A503S^, or Hsp104^DPLA-DWB^ plus or minus Hsp40 and Hsp70, as indicated, for 24 h at 37°C. Wells were then stained with crystal violet to visualize biomass. Images shown depict wells that are all from a single trial (top). Wells from B were resuspended and quantified (bottom). Biomass for each condition was compared to control treatment with buffer using a two-tailed, unpaired t-test. (*N* ≥ 4 biological replicate trials, with two technical replicate wells for each trial, bars show mean ± SEM, *P < 0.05, ****P < 0.0001, and ns *P* > 0.05). (**D**) Experiments were performed as in C using SH1000 Δ*αβpsm S. aureus*. Following treatment, wells were stained with crystal violet to visualize biomass (top) and then resuspended and quantified (bottom). Biomass for each condition was compared to control treatment with buffer using a two-tailed, unpaired t-test. (*N* ≥ 4 biological replicate trials, with three technical replicate wells for each trial, bars show mean ± SEM, and all *P* values were greater than 0.05).

We next sought to investigate if potentiated Hsp104 variants could drive the disassembly of complex biofilm matrices. To do so, we grew *S. aureus* SH1000 in microtiter plates and then treated these biofilms with purified Hsp104, Hsp104^A503S^, or Hsp104^A503S^ supplemented with co-chaperones Hsp40 and Hsp70 (Fig. 6A). To validate our approach, we first compared biofilm formation between *S. aureus* SH1000 and SH1000 Δ*αβpsm*, which cannot produce PSMα or PSMβ peptides (8). As a positive control for biofilm disruption, we used Proteinase K, which has been shown to disrupt biofilms by non-specific digestion of proteins (8). Following treatment with buffer or Proteinase K for 24 h, we stained the biofilms with crystal violet dye, photographed the residual material, and then resuspended and quantified the signal (Fig. 6A and B). We find that treatment with Proteinase K decreases biofilm biomass produced by SH1000 *S. aureus* by approximately 50%. The Δ*αβpsm* strain decreased biomass formation by approximately 25%. As expected, treatment of the Δ*αβpsm* strain with Proteinase K does not further decrease biofilm biomass. Next, we treated the SH1000 biofilms with Hsp104 (Fig. 6C). Treatment with the chaperones Hsp40 and Hsp70 alone subtly decreased biomass, but this effect was not significant. Treatment with Hsp104^WT^ had no effect on biomass while Hsp104^A503S^ had a subtle but non-statistically significant effect on the biofilm. However, treatment with Hsp104^A503S^ disaggregase in combination with its co-chaperones Hsp40 and Hsp70 drives a visible decrease in biomass, corresponding to a 25% reduction in biofilm biomass (Fig. 6C). As a negative control, we also treated SH1000 biofilms with Hsp104^Y257A-E285Q-Y662A-E687Q^ (Hsp104^DPLA-DWB^), an ATPase dead, substrate-binding deficient mutant (18). This variant also had no effect on biofilm biomass. Finally, to confirm that the changes we observe are specific to disruption of the PSM component, we treated the Δ*αβpsm* strain with Hsp104 (Fig. 6D). As expected, we see no significant change in biofilm biomass upon treatment of these materials with Hsp104 or the potentiated Hsp104^A503S^ variant complemented with Hsp40 and Hsp70. We therefore conclude that Hsp104^A503S^ can disrupt preformed *S. aureus* biofilm matrices, which validates use of our yeast model system as a useful tool for identifying biofilm modulators and provides further support for the idea that disruption of the proteinaceous component of *S. aureus* biofilms can destabilize them sufficiently to allow for removal.

## DISCUSSION

Biofilms are poorly defined heterogeneous mixtures that are challenging to study using conventional approaches (1, 4, 7). Here, we demonstrate that the aggregation of *S. aureus* biofilm-associated proteins can be studied by use of a simple yeast model system. We have established that this system can be used as a platform for studying the toxicity, aggregation, and solubility of PSMα peptides in a genetically tractable manner. We find that PSMα toxicity and solubility in yeast recapitulate known features of these peptides. Expression of the PSM peptides also leads to the accumulation of unusual spherical vesicle-like structures, presumably due to the surfactant character of the peptides. These structures resemble the extracellular vesicles that are secreted by *S. aureus*, which is a process that relies upon the presence of PSMα peptides (49). Using this system, we have identified a key position where substitution of glycine for threonine correlates with key differences noted in PSMα peptide properties. Finally, we have demonstrated that variants of the AAA+ disaggregase Hsp104 are capable of suppressing the toxicity and aggregation of the PSMα peptides and that they are further able to destabilize preformed biofilm matrices to facilitate their removal. It is important to note that Hsp104^A503S^ was originally isolated in a screen against the ALS-associated protein TDP-43, so it has not been substrate-optimized to disrupt the PSMα proteins (55). Thus in the future, it will be important to further boost the disaggregase activity of Hsp104^A503S^ against the PSMα peptides through protein engineering. This tunable activity and dissolution without proteolysis represent key benefits to this strategy. Our work suggests that further efforts to develop and apply potentiated Hsp104 variants may be a useful strategy for disrupting heterogeneous biofilm matrices, including those of diverse bacteria beyond just those of *S. aureus*.

We have employed this newly developed yeast model system to define key differences between PSMα1, PSMα2, and PSMα4, which have relatively similar sequences but distinct properties. We find that a single missense substitution of glycine for threonine at position 6 enhances the toxicity and insolubility of PSMα1 and PSMα2, while in the PSMα4 sequence, substitution of threonine for glycine has the opposite effect. We hypothesize that these effects are due to threonine increasing the steric zipper content of this region, which is supported by our ZipperDB calculations. These findings support the relevance of using this simple model system as a platform for studying the drivers of host toxicity and aggregation of biofilm-associated proteins. It may be informative to use this system to explore the full sequence space of these peptides to comprehensively map the drivers of PSM aggregation. Such an approach could overcome inherent limitations when studying a small subset of variant peptides using traditional approaches that are reliant on solid-phase peptide synthesis. Furthermore, we anticipate that this yeast model system could be a broadly useful platform for screening for modulators of PSMα protein aggregation that could overcome many of the limitations of current approaches for assaying biofilm disruption.

Disruption of biofilms is a key goal for healthcare, water treatment, and other applications (1 - 4). Just as the amyloid component of PSMα peptides stabilizes biofilms and makes them resilient, disruption of this component could destabilize biofilm matrices and make their removal more straightforward. Our results indicate that Hsp104^A503S^ disrupts preformed biofilm matrices and facilitates their removal, suggesting that in practice, purified Hsp104 variants could be applied to surfaces to disrupt the amyloid component, allowing for the removal of the biofilm under mild conditions. Based on the results presented in this study, the currently in-hand Hsp104 variants may be sufficiently active for use in this context. However, we do note incomplete clearance of the vesicle structures and only a 25% reduction in biofilm biomass, though this reduction is of a similar magnitude to that achieved by genetic deletion of PSMα and PSMβ. In the future, it may be necessary to further engineer Hsp104 for this particular application. Further, because biofilms are comprised of multiple components that collectively promote adherence, including polysaccharides and DNA, it may be important to disrupt additional components of the biofilm to allow for complete removal. Should further engineering of Hsp104 be necessary, we envision incorporating our yeast model into an approach similar to those used for isolating enhanced Hsp104 variants that counter TDP-43, FUS, and α-syn toxicity (22 - 55 - 55). In sum, we have demonstrated that this new yeast model system can be used to study and uncover key features of the amyloid component of biofilms and that Hsp104 disaggregases can be applied as a novel tool for biofilm disruption and removal.

## SUPPLEMENTAL MATERIAL

FIG S1 mbio.00587-23-s0001.tif PSMα peptides expressed without a GFP tag are toxic in yeast. BYΔ*hsp104* yeast were transformed with the indicated 423GAL-PSMα plasmid or 423GAL empty vector control. Strains were serially diluted fivefold and spotted on glucose (off) or galactose (on) media. Toxicity trends without the GFP tag were similar to the trends with the plasmids with a C-terminal GFP tag.

FIG S2 mbio.00587-23-s0002.tif Further characterization of strains from Figure 2. (A) w303Δ*hsp104* strains were integrated with two copies of the indicated PSMα peptide or vector control using the 303GAL-PSMα-GFP and 304GAL-PSMα-GFP vectors. Strains were serially diluted fivefold and spotted on glucose (off) or galactose (on) media. (B) Strains from A were induced for 5 h, lysed, and immunoblotted. 3-Phosphoglycerate kinase (PGK) serves as a loading control. (C) Strains from A were induced for 15 h and stained with CellTracker Blue CMAC (7-amino-4-chloromethylcoumarin) to visualize the vacuoles. Scale bar = 5 microns. (D-F) Puncta (PSMα1) or vesicle (PSMα2-4) circularity, area, and perimeter were calculated from images as acquired in Fig 2D following 5 h induction. Values were compared to each other using a one-way ANOVA with Tukey’s multiple comparisons test (N≥100, individual points shown as dots, bars show means, *p<0.05 and ****p<0.0001).

FIG S3 mbio.00587-23-s0003.tif Potentiated Hsp104 variants do not suppress the toxicity of PSMα1 and subtly suppress PSMα2 toxicity when PSMα2 is expressed at low levels. (A) w303Δ*hsp104* yeast was transformed with 423GAL-PSMα1-GFP and 425GAL-PSMα1-GFP and then subsequently transformed with the indicated 416GAL-Hsp104 variant or vector control. Strains were serially diluted fivefold and spotted on glucose (off) or galactose (on) media. (B) Strains from A were induced for 5 h, lysed, and immunoblotted. Uninduced cells serve as a loading control. 3-Phosphoglycerate kinase (PGK) serves as a loading control. (C) w303Δ*hsp104* yeast was transformed with 303GAL-PSMα2-GFP and 304GAL-PSMα2-GFP and then subsequently transformed with the indicated 416GAL-Hsp104 variant or vector control. Spotting assays were performed as in A. (D) Strains from C were processed for immunoblotting as in B. Results with 423GAL-PSMα2-GFP and 424GAL-PSMα2-GFP are shown in Figure 4.

ASM does not own the copyrights to Supplemental Material that may be linked to, or accessed through, an article. The authors have granted ASM a non-exclusive, world-wide license to publish the Supplemental Material files. Please contact the corresponding author directly for reuse.
