# Supplementary figures and images for: Probing the drivers of Staphylococcus aureus biofilm protein amyloidogenesis and disrupting biofilms with engineered protein disaggregases
Source: mBio. 2023 May 17;14(4):e00587-23. doi: 10.1128/mbio.00587-23 (PMC10470818; doi:10.1128/mbio.00587-23)

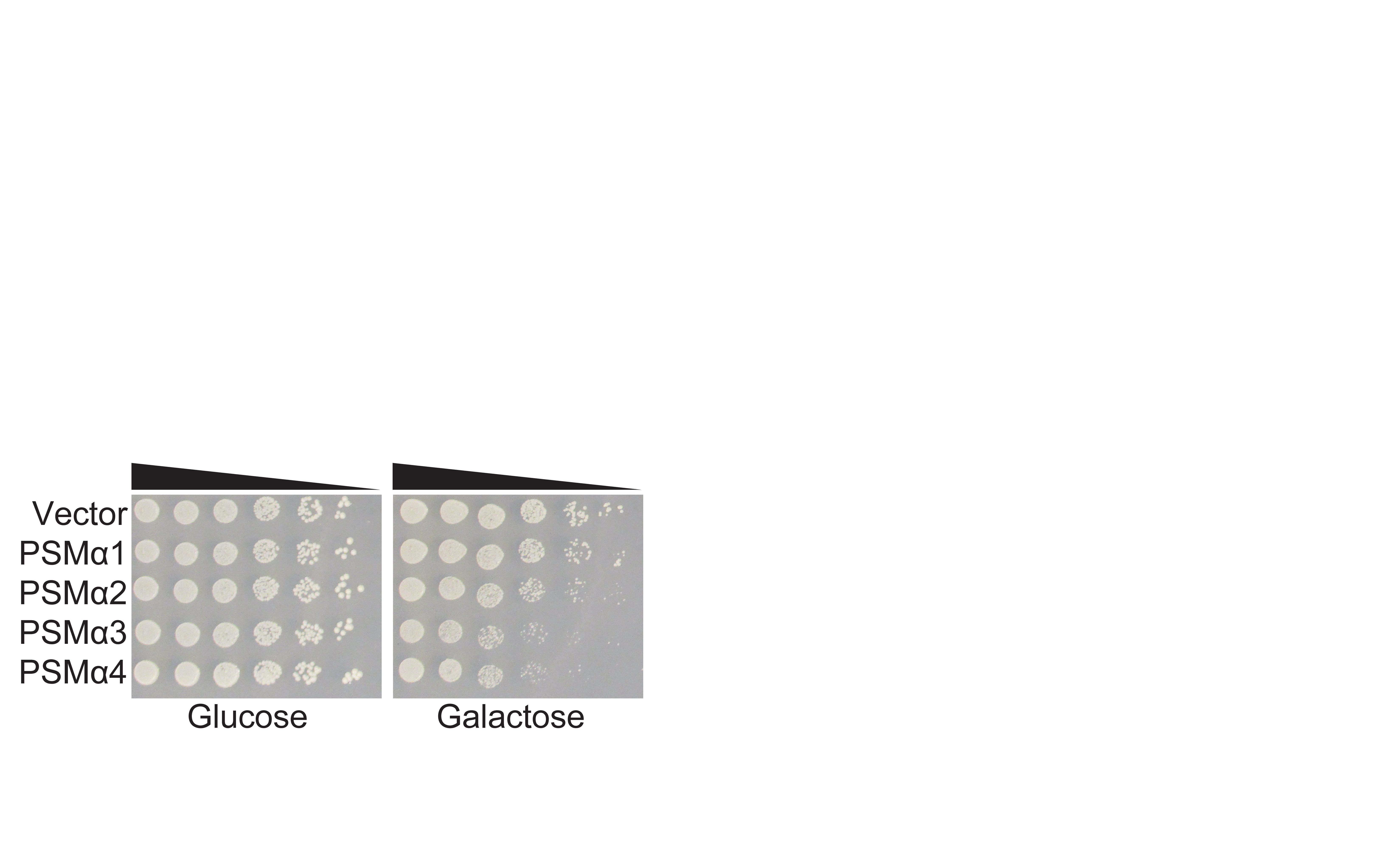

Supplement: FIG S1 — PSMα peptides expressed without a GFP tag are toxic in yeast. BYΔhsp104 yeast were transformed with the indicated 423GAL-PSMα plasmid or 423GAL empty vector control. Strains were serially diluted fivefold and spotted on glucose (off) or galactose (on) media. Toxicity trends without the GFP tag were similar to the trends with the plasmids with a C-terminal GFP tag. [file mbio.00587-23-s0001.tif]

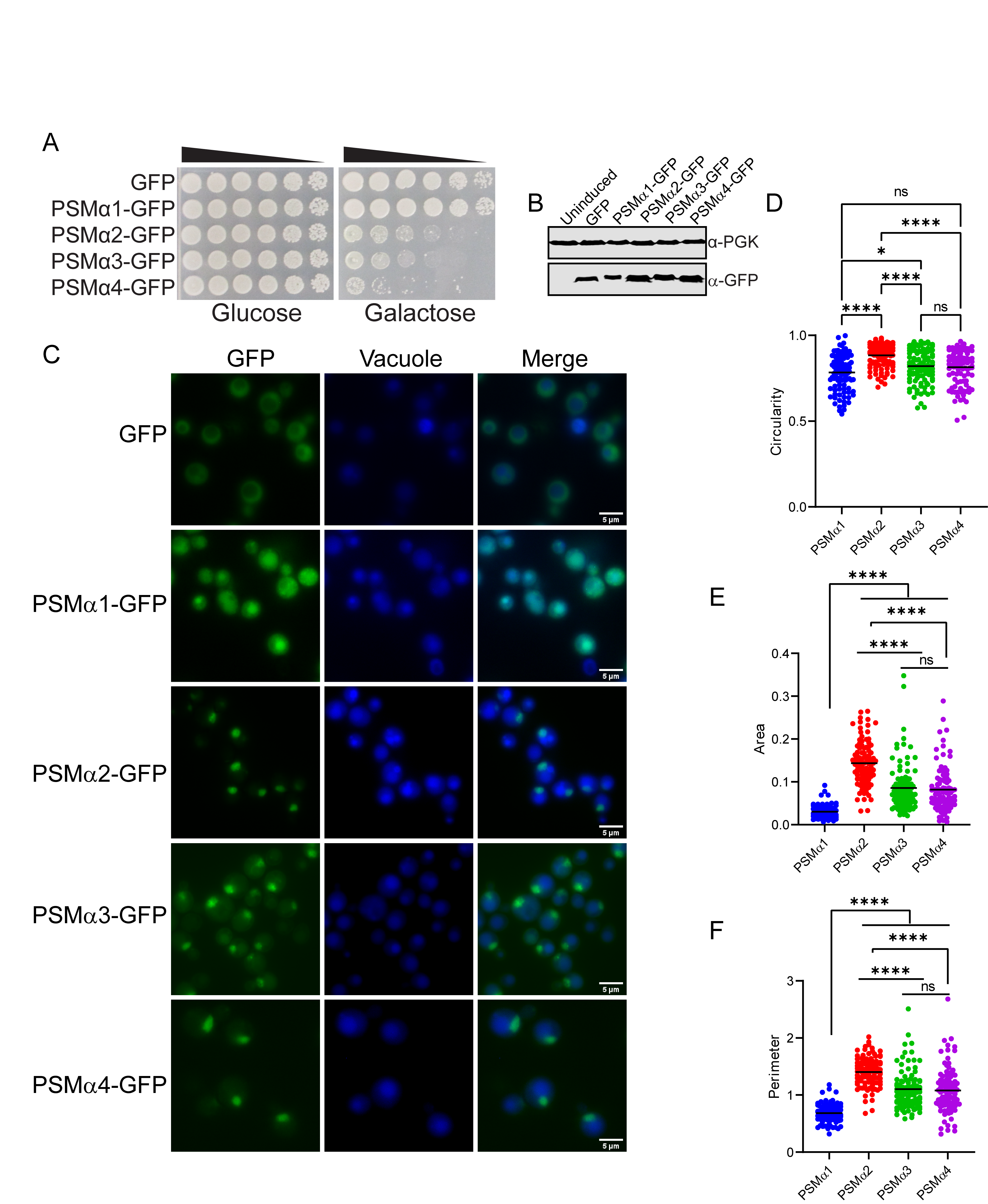

Supplement: FIG S2 — Further characterization of strains from Figure 2. (A) w303Δhsp104 strains were integrated with two copies of the indicated PSMα peptide or vector control using the 303GAL-PSMα-GFP and 304GAL-PSMα-GFP vectors. Strains were serially diluted fivefold and spotted on glucose (off) or galactose (on) media. (B) Strains from A were induced for 5 h, lysed, and immunoblotted. 3-Phosphoglycerate kinase (PGK) serves as a loading control. (C) Strains from A were induced for 15 h and stained with CellTracker Blue CMAC (7-amino-4-chloromethylcoumarin) to visualize the vacuoles. Scale bar = 5 microns. (D-F) Puncta (PSMα1) or vesicle (PSMα2-4) circularity, area, and perimeter were calculated from images as acquired in Fig 2D following 5 h induction. Values were compared to each other using a one-way ANOVA with Tukey’s multiple comparisons test (N≥100, individual points shown as dots, bars show means, *p<0.05 and ****p<0.0001). [file mbio.00587-23-s0002.tif]

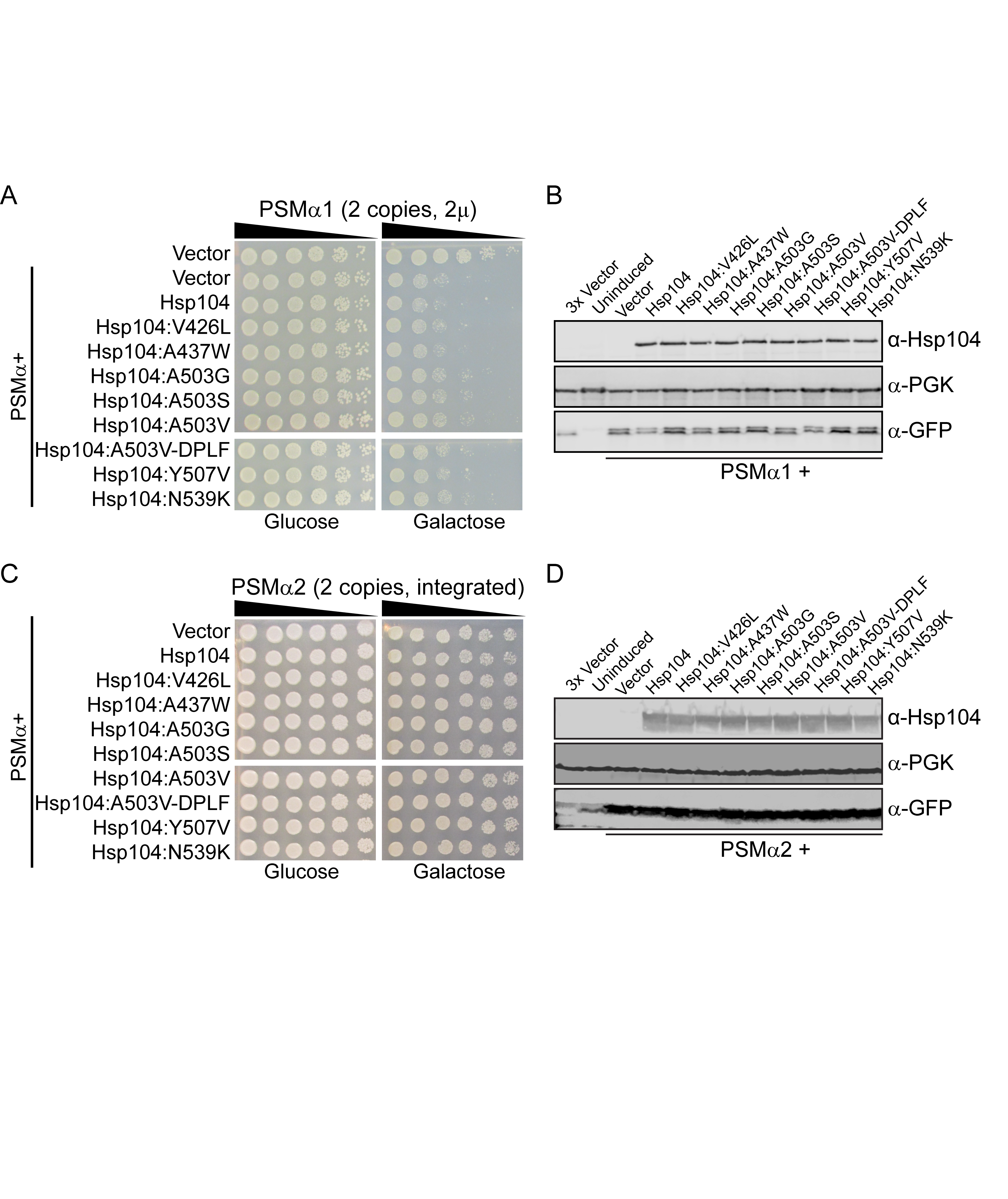

Supplement: FIG S3 — Potentiated Hsp104 variants do not suppress the toxicity of PSMα1 and subtly suppress PSMα2 toxicity when PSMα2 is expressed at low levels. (A) w303Δhsp104 yeast was transformed with 423GAL-PSMα1-GFP and 425GAL-PSMα1-GFP and then subsequently transformed with the indicated 416GAL-Hsp104 variant or vector control. Strains were serially diluted fivefold and spotted on glucose (off) or galactose (on) media. (B) Strains from A were induced for 5 h, lysed, and immunoblotted. Uninduced cells serve as a loading control. 3-Phosphoglycerate kinase (PGK) serves as a loading control. (C) w303Δhsp104 yeast was transformed with 303GAL-PSMα2-GFP and 304GAL-PSMα2-GFP and then subsequently transformed with the indicated 416GAL-Hsp104 variant or vector control. Spotting assays were performed as in A. (D) Strains from C were processed for immunoblotting as in B. Results with 423GAL-PSMα2-GFP and 424GAL-PSMα2-GFP are shown in Figure 4. [file mbio.00587-23-s0003.tif]
